# Protein tyrosine phosphatase receptor type R is required for Purkinje cell responsiveness in cerebellar long-term depression

**DOI:** 10.1186/s13041-014-0092-8

**Published:** 2015-01-09

**Authors:** Mirthe Erkens, Keiko Tanaka-Yamamoto, Guy Cheron, Javier Márquez-Ruiz, Cynthia Prigogine, Jan TG Schepens, Nael Nadif Kasri, George J Augustine, Wiljan JAJ Hendriks

**Affiliations:** Department of Cell Biology, Radboud Institute for Molecular Life Sciences, Radboud University Medical Centre, PO Box 9101, Nijmegen, 6500 HB The Netherlands; Center for Functional Connectomics, Korea Institute of Science and Technology, 39-1 Hawolgokdong, Seongbukgu, Seoul, 136-791 Republic of Korea; Laboratory of Electrophysiology, Université de Mons, Mons, 7000 Belgium; Laboratory of Neurophysiology and Movement Biomechanics, CP601, ULB Neurosciences Institut, Université Libre de Bruxelles, Brussels, 1070 Belgium; División de Neurociencias, Universidad Pablo de Olavide, Sevilla, 41013 Spain; Department of Cognitive Neurosciences, Nijmegen, 6500 HB The Netherlands; Department of Human Genetics, Donders Institute for Brain, Cognition and Behaviour, Radboud University Medical Centre, PO Box 9101, Nijmegen, HB 6500 The Netherlands; Lee Kong Chian School of Medicine, Nanyang Technological University, Singapore, Singapore; Institute of Molecular and Cell Biology, 61 Biopolis Drive, Proteos, Singapore, 138673 Singapore

**Keywords:** Cerebellum, ERK, Long-term synaptic plasticity, Knock-out mice, LTD, PTPBR7, PTP-SL

## Abstract

**Background:**

Regulation of synaptic connectivity, including long-term depression (LTD), allows proper tuning of cellular signalling processes within brain circuitry. In the cerebellum, a key centre for motor coordination, a positive feedback loop that includes mitogen-activated protein kinases (MAPKs) is required for proper temporal control of LTD at cerebellar Purkinje cell synapses. Here we report that the tyrosine-specific MAPK-phosphatase PTPRR plays a role in coordinating the activity of this regulatory loop.

**Results:**

LTD in the cerebellum of *Ptprr*^−/−^ mice is strongly impeded, *in vitro* and *in vivo*. Comparison of basal phospho-MAPK levels between wild-type and PTPRR deficient cerebellar slices revealed increased levels in mutants. This high basal phospho-MAPK level attenuated further increases in phospho-MAPK during chemical induction of LTD, essentially disrupting the positive feedback loop and preventing α-amino-3-hydroxy-5-methyl-4-isoxazolepropionic acid receptor (AMPAR) phosphorylation and endocytosis.

**Conclusions:**

Our findings indicate an important role for PTPRR in maintaining low basal MAPK activity in Purkinje cells. This creates an optimal ‘window’ to boost MAPK activity following signals that induce LTD, which can then propagate through feed-forward signals to cause AMPAR internalization and LTD.

## Background

**Figure 1:**
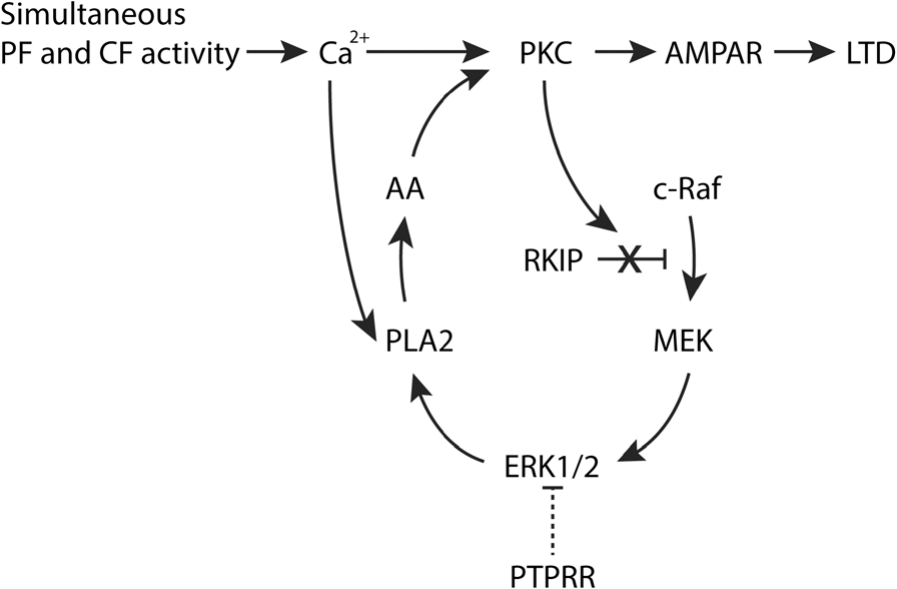
**Positive feedback loop model underlying cerebellar LTD.** Abbreviations are explained in the text. Adapted from [13].

Neuronal signaling in the brain depends on dynamic changes in the strength of synaptic transmission. One of the major mechanisms that regulate synaptic strength involves the regulated trafficking of α-amino-3-hydroxy-5-methyl-4-isoxazolepropionic acid receptors (AMPARs) into and out of synapses. Increases in synaptic AMPARs result in long-term potentiation of synaptic strength and AMPAR removal in long-term depression (LTD) [1,2]. In the cerebellum, Purkinje cells (PCs) represent the key output cells that respond to excitatory synaptic signals coming from both parallel fibers (PFs) and climbing fibers (CFs) [3,4]. LTD at the PC synapse occurs upon simultaneous firing of PF and CF [5,6]. CF activity causes a Ca^2+^ influx through voltage-gated ion channels and sensitizes the PC for the IP_3_ that is produced due to PF stimuli. Collectively, the PF and CF signals lead to Ca^2+^ release from the ER of PCs [7-12]. The transient change in local Ca^2+^ concentration activates a positive feedback loop that transforms the initial transient signal into a long-term response that can last for hours or longer [13]. Computational predictions [14,15] and recent experimental studies in cerebellar slices [13,16] have established the key molecular players within this feedback loop (Figure 1). Briefly, the Ca^2+^ levels that occur during LTD induction activate protein kinase C (PKC), which in turn phosphorylates the Raf kinase inhibitory protein (RKIP). In the unphosphorylated state, RKIP sequesters c-Raf thereby preventing its interaction with and activation of MEK (Mitogen-Activated Protein Kinase/Extracellular Signal-Regulated Kinase Kinase) [17,18]. PKC-mediated RKIP phosphorylation thus triggers the sequential activation of the kinase MEK and its downstream target, the MAPK ERK. This leads to phospholipase A2 (PLA2) activation [19] and subsequent arachidonic acid (AA) release [20], which closes the positive feedback loop by direct binding and activation of PKC [21,22]. The resulting sustained activation of PKC at the cell membrane translates into phosphorylation of AMPARs and their subsequent internalization [23], effectively causing the depressed PC response to PF input that is known as LTD. Aberrations in the above signaling steps or in the control of intracellular Ca^2+^ levels would be expected to impair LTD regulation and malfunction of PC output. Indeed, deficiencies in calcium-binding proteins - such as calretinin, calbindin, and parvalbumin - disturb PC firing [24] and impair LTD. However, our knowledge of the molecular players that regulate the components of the signaling feedback loop that leads to AMPAR internalization and cerebellar LTD is still limited.

One of the key players within the LTD feedback loop is ERK1/2, which is activated by dual phosphorylation on a specific pair of tyrosine (Thr-202) and threonine (Tyr-204) residues within the activation loop [25]. MAPK inhibition can be mediated by phosphatases that dephosphorylate specifically phosphoserine and/or phosphothreonine residues [26,27]. Some of these phosphatases [28,29] are especially suited to selectively bind and inactivate MAPKs because of the presence of a kinase interaction motif (KIM) just N-terminal of their phosphatase domain [30]. This KIM-mediated interaction not only results in MAPK dephosphorylation and inactivation, it also mediates sequestration of the MAPK, hence preventing its translocation to other cellular compartments [31-34]. Notably, the protein tyrosine phosphatase receptor-type R (PTPRR) has been linked to cerebellar function [35]. PTPRR expression levels are highest in PCs [36] and PTPRR-deficient mice exhibit cerebellum-associated defects in fine motor coordination and balance skills [37] as well as an altered exploratory behavior [38]. PTPRR preferentially interacts with ERK1/2 [32,39-41] and PTPRR-deficient mice have increased ERK1/2 phosphorylation in their brain tissue [37].

We hypothesized that PTPRR may be involved in regulating cerebellar LTD in the way depicted by the dashed line in Figure 1. To test this hypothesis, we examined the electrophysiological and molecular consequences of PTPRR deficiency for the process of LTD *in vitro* and *in vivo.* In cerebellar slices from *Ptprr*^*−/−*^ mice we observed significantly elevated ERK1/2 phospholevels under basal conditions, and the absence of LTD associated with loss of the increase in phosphorylated ERK1/2 and AMPAR normally associated with LTD induction. In addition, cerebellar LTD was strongly impeded when measured in awake *Ptprr*^*−/−*^ mice. We conclude that PTPRR allows optimal functioning of the positive feedback loop by maintaining low basal ERK1/2 phosphorylation levels, thereby yielding PC responsiveness to LTD-inducing stimuli.

## Results

### LTD in cerebellar slices is regulated by PTPRR

**Figure 2:**
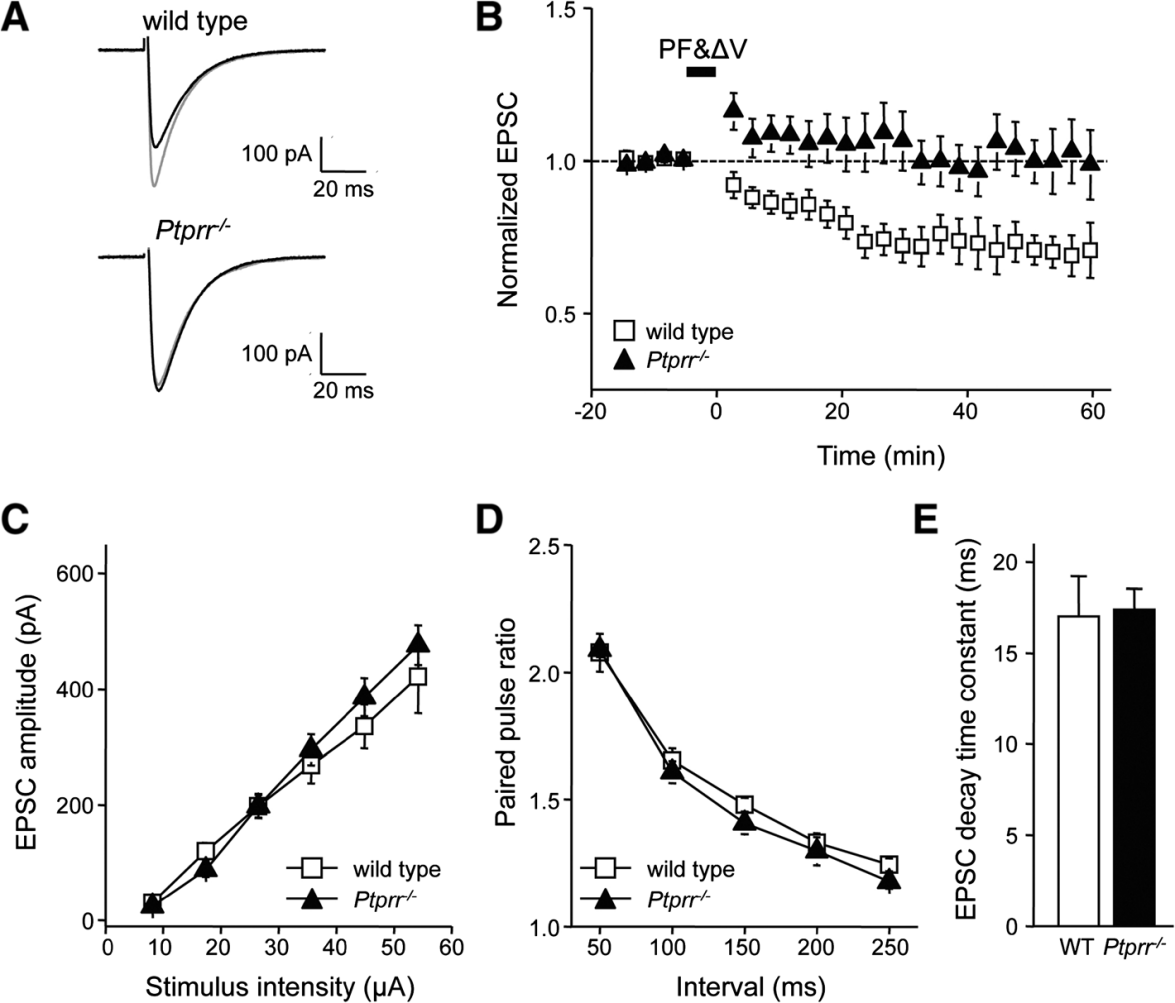
**The impairment of cerebellar LTD at synapses between parallel fiber and Purkinje cells in** ***Ptprr*** ^**−/−**^ **mice. (A)** Superimposed PF-EPSCs recorded before (gray line) and 20 min after (black line) PF&ΔV in PCs of wild-type or *Ptprr* ^−/−^ cerebellar slices. **(B)** Averaged time course of LTD triggered by PF&ΔV. The LTD was recorded from PCs in wild-type (n = 9) or *Ptprr* ^−/−^ (n = 9) cerebellar slices. PF-EPSC amplitudes are normalized to their mean prestimulation level. **(C)** Amplitudes of PF-EPSCs elicited by PF stimuli of increasing intensity. **(D)** PPF ratios of PF-EPSCs elicited by a pair of PF stimuli with different intervals. **(E)** Decay time constant of PF-EPSC. Error bars represent SEM.

To test the involvement of PTPRR in cerebellar LTD, we stimulated PF synapses while recording excitatory postsynaptic currents (PF-EPSCs) from PCs in wild-type and *Ptprr*^−/−^ cerebellar slices. While pairing electrical stimulation of PFs together with PC depolarization at 1 Hz for 5 min (PF&ΔV) did induce LTD in the wild-type PCs, PF&ΔV failed to do so in *Ptprr*^−/−^ PCs (Figure 2A, B). The reduction of PF-EPSC calculated at 30–40 min after PF&ΔV was significantly smaller in the *Ptprr*^−/−^ PCs than in the wild-type PCs (wild-type: 28.3 ± 6.3% (n = 9), *Ptprr*^−/−^: 1.6 ± 7.2% (n = 9), p < 0.05, Student’s *t*-test).

The PTPRR deficiency may have elevated basal MAPK activity [37] in PCs, thereby reducing PF-induced EPSCs and preventing PF&ΔV from triggering LTD in the *Ptprr*^−/−^ slices. To consider this possibility, we measured PF-EPSCs in PCs of wild-type and *Ptprr*^−/−^ cerebellar slices at different stimulus intensities. The relationship between PF-EPSC amplitude and stimulus intensity was linear and not significantly different between wild-type and *Ptprr*^−/−^ PCs (Figure 2C; p = 0.36, two-way ANOVA; n = 10 for wild type, n = 12 for *Ptprr*^*−/−*^). This indicates that loss of PTPRR caused no change in the basal transmission at PF-PC synapses. The paired-pulse facilitation (PPF) ratios were also unaltered in *Ptprr*^*−/−*^ PCs compared with wild-type PCs (Figure 2D; p = 0.1, two-way ANOVA; n = 14 for wild type, n = 12 for *Ptprr*^*−/−*^), indicating that the probability of glutamate release from presynaptic PF terminals is not affected in *Ptprr*^*−/−*^ mice. Furthermore, we determined the PF-EPSC decay time constant in wild-type and *Ptprr*^−/−^ slices as a measure of AMPAR functional properties [42]. EPSC decay was found to be identical in wild-type and *Ptprr*^−/−^ PCs (Figure 2E; p = 0.88, Student’s *t*-test; n = 10 for wild-type, n = 12 for *Ptprr*^*−/−*^), suggesting that AMPAR composition is unaltered in *Ptprr*^−/−^ slices. Thus, while the efficacy of basal synaptic transmission and AMPAR performance is unaltered in the PCs lacking PTPRR, expression of LTD is impaired. These findings suggest a specific role for PTPRR in the regulation of cerebellar LTD.

### PTPRR determines the dynamic range of ERK phosphorylation during cerebellar LTD

**Figure 3:**
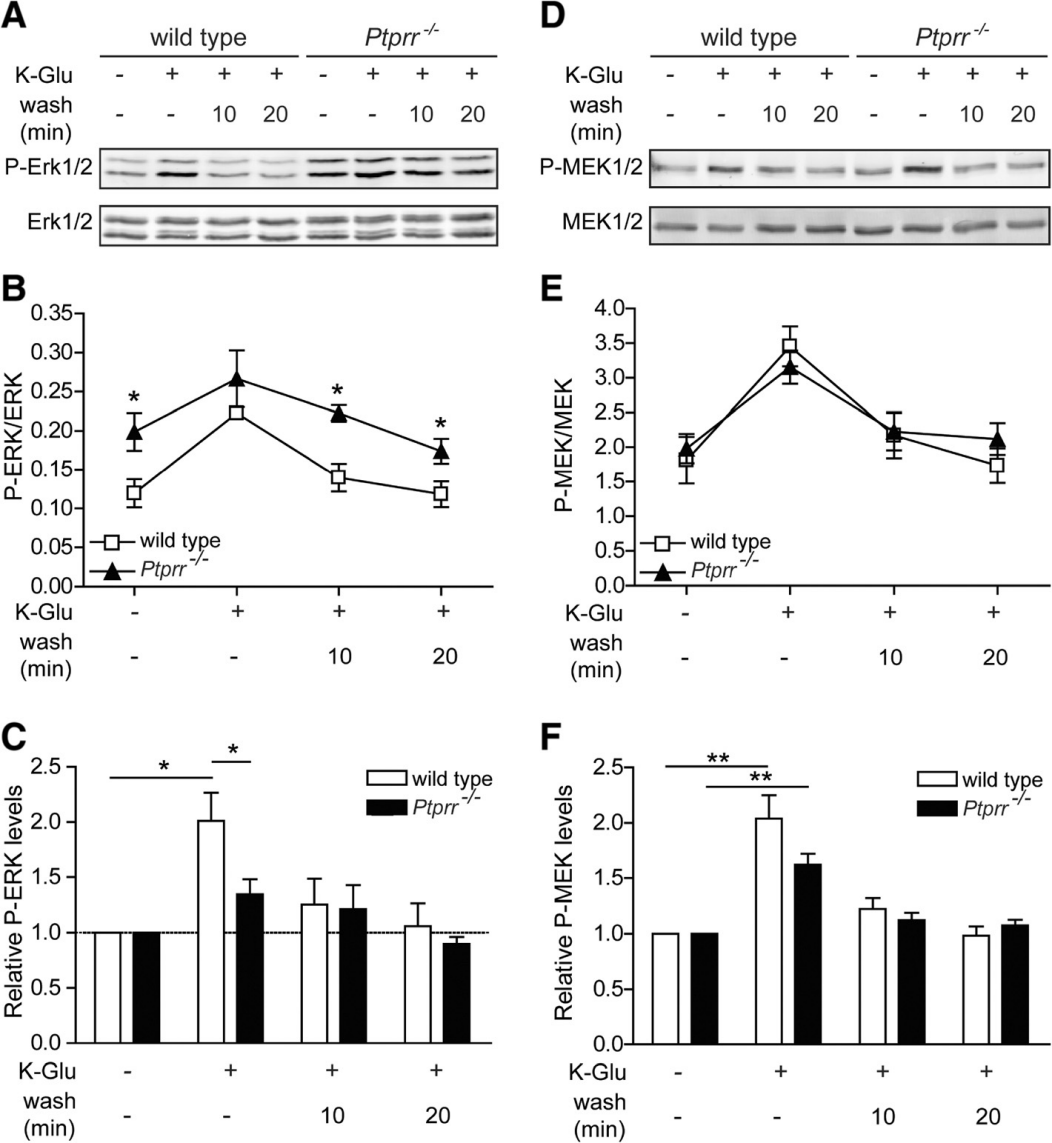
**ERK and MEK phosphorylation profiles following LTD induction in wild type and** ***Ptprr*** ^**−/−**^ **cerebellar slices. (A)** Immunoblot detecting phospho-ERK1/2 (upper panel) and total ERK1/2 (lower panel) in cerebellar slices of wild-type and *Ptprr* ^−/−^ mice under basal conditions, after a 5-min stimulation with K-Glu to induce LTD, and following subsequent 10 or 20 min washing out of K-Glu. Representative images are shown. **(B)** Quantitative representation of phospho-ERK1/2 over total ERK1/2 levels (arbitrary units) in lysates of cerebellar slices as determined under (A). Results are presented as mean values and error bars indicate SEM from five independent experiments (Student’s *t*-test, *p < 0.05). **(C)** An alternative quantitative representation of the data determined under (A) showing the fold increase of phospho-ERK1/2-total ERK1/2 ratios in the different samples as compared to the ratio under basal conditions. Results are presented as mean values and error bars indicate SEM from five independent experiments (Student’s *t*-test, *p < 0.05). **(D)** Immunoblot detecting phospho-MEK1/2 (upper panel) and total MEK1/2 (lower panel) in cerebellar slices of wild-type and *Ptprr* ^−/−^ mice under basal conditions, stimulated with K-Glu to induce LTD, or after subsequent wash out of K-Glu for 10 or 20 min. Representative images out of five independent experiments are shown. **(E)** Quantification of the ratio of phospho-MEK1/2 over total MEK1/2 levels (arbitrary units) from the cerebellar slice lysates as described under (D). Results are presented as mean values and error bars indicate SEM from five independent experiments. **(F)** Quantitative representation of the fold increase of relative phospho-MEK1/2 levels compared to the relative levels under basal conditions as determined in the cerebellar slice lysates described under (D). Results are presented as mean values and error bars indicate SEM from five independent experiments (Student’s *t*-test, **p < 0.01).

To determine whether the ability of PTPRR to inhibit MAPK activity [37] is indeed relevant for cerebellar LTD, we assessed ERK1/2 activity levels before and after LTD in wild-type and *Ptprr*^−/−^ cerebellar slices (Figure 3A). We used a chemical method to induce LTD [13,43-45]: this involved treating cerebellar slices with a solution containing high K^+^ and glutamate (K-Glu) to mimic the simultaneous CF-induced membrane depolarization and intracellular increase of Ca^2+^ and the PF-induced mGluR activation that underlies LTD. This step was followed by subsequent washes of 10 or 20 minutes to evaluate the effect of removal of the LTD-inducing agent.

In wild-type cerebellar slices, K-Glu treatment elevated phospho-ERK1/2 levels, which returned to basal levels after washout of the chemical stimulants (Figure 3A-C). Under basal conditions, phospho-ERK1/2 levels were significantly elevated in the cerebellar slices of *Ptprr*^−/−^ mice as compared to wild-type slices (p < 0.03, Student’s *t*-test, n = 5; Figure 3B), which is in line with earlier observations in total cerebella of these mice [37]. Elevated phospho-ERK1/2 levels were also detected in the K-Glu-treated *Ptprr*^−/−^ slices after subsequent washes (10 min wash: p < 0.004, 20 min wash: p < 0.04, Student’s *t*-test, n = 5). Directly after the K-Glu treatment, however, phospho-ERK1/2 levels were comparable in wild-type and *Ptprr*^−/−^ cerebellar slices. This indicates that the net increase in ERK1/2 activity is much lower in *Ptprr*^−/−^ slices than in wild-type slices following chemical induction of LTD.

To visualize this more directly, the ratio of phospho-ERK1/2 over total ERK1/2 was determined and normalized to the ratio under basal conditions (Figure 3C). K-Glu treatment resulted in a two-fold increase in phospho-ERK1/2 levels in wild-type slices (p < 0.02, one sample *t*-test, n = 5), which returned to basal levels after washout of the K-Glu solution. The net increase in phospho-ERK1/2 in response to K-Glu treatment was significantly less in *Ptprr-/-* slices compared with wild-type slices (p < 0.05, Student’s *t*-test, n = 5) and, importantly, was not significantly different compared to basal levels. Based on these findings, we propose that high basal phospho-ERK1/2 levels in *Ptprr*^−/−^ cerebellar slices occlude a significant increase of ERK1/2 activity upon LTD induction. Thus, in wild-type conditions PTPRR serves to provide an optimal window for ERK activation by ensuring low basal phosphorylation of ERK1/2.

### PTPRR does not affect events upstream of MAPK in the positive feedback loop

To determine whether PTPRR deficiency causes additional deregulation in the positive feedback cycle that controls cerebellar LTD, we next evaluated MEK1/2 activity levels in K-Glu-treated *Ptprr*^−/−^ and wild-type cerebellar slices. MEK1/2 is an upstream activator of ERK1/2 (Figure 1) and is activated when phosphorylated at residues Ser-218 and Ser-222. In contrast to what was found for ERK1/2, PTPRR deficiency had no effect on phospho-MEK1/2 levels in the cerebellar slices. Under all conditions tested, phospho-MEK1/2 levels were equal in *Ptprr*^−/−^ and wild-type slices and in both cases a significant increase in phospho-MEK levels was discernble upon LTD induction (wild-type: p < 0.008, *Ptprr*^−/−^: p < 0.003, one sample *t*-test, n = 5). Phospho-MEK levels returned to basal levels after washing out the LTD-inducing solution (Figure 3D-F). These results are in line with PTPRR acting at the level of MAPK in the regulatory signaling network, rather than at upstream targets. We thus hypothesize that the absence of LTD in PTPRR-deficient cerebellar slices results from the inability to translate increased MEK1/2 activity into further activation of the LTD regulatory loop, due to insufficient stimulus-induced increases in ERK1/2 phosphorylation. As a consequence, AMPAR internalization and thus the induction of LTD will be precluded.

### PTPRR is required for GluA2-S880 phosphorylation during LTD induction

**Figure 4:**
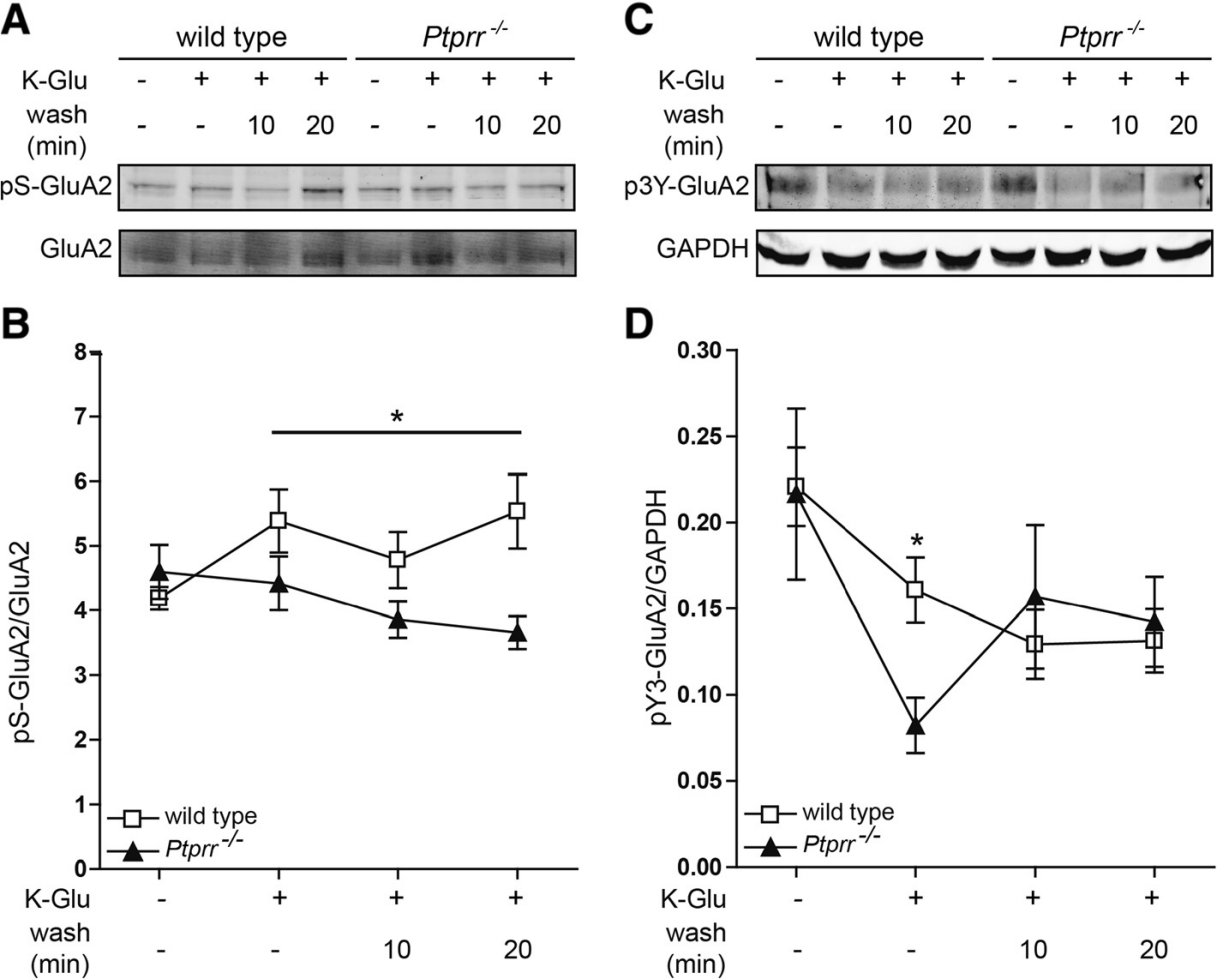
**Phosphorylation on GluA2-S880, but not on GluA2-Y869/Y873/Y876, is impaired in** ***Ptprr*** ^**−/−**^ **cerebellar slices upon LTD-induction. (A)** Immunoblot detecting phospho-S880-GluA2 (upper panel) and total GluA2 (lower panel) in cerebellar slices of wild-type and *Ptprr* ^−/−^ mice under basal conditions, stimulated with K-Glu to induce LTD, or after subsequent wash out of K-Glu for 10 or 20 min. **(B)** Quantitative representation of the relative phospho-GluA2 levels, calculated as the ratio of phospho-GluA2 over total GluA2 levels (arbitrary units) from the different cerebellar slice lysates as described under (A). Results are presented as mean values and error bars indicate SEM from five independent experiments (ANOVA repeated measures, *p < 0.05). **(C)** Phospho-Y869/Y873/Y876-GluA2 (upper panel) and GAPDH (lower panel) immunoblot detection in wild-type and *Ptprr* ^−/−^ cerebellar slices under basal conditions, stimulated with K-Glu to induce LTD, or after subsequent washes of 10 or 20 min. **(D)** Quantitative representation of the relative p3Y-GluA2 levels, normalized over GAPDH levels (arbitrary units) from the different cerebellar slice lysates as described under (C). Results are presented as mean values and error bars indicate SEM from four independent experiments (ANOVA repeated measures, *p < 0.02).

PKC-mediated phosphorylation of the S880 residue on the GluA2 subunit is known to trigger AMPAR internalization by endocytosis to depress synaptic transmission during LTD induction [23]. To test whether PTPRR deficiency affects this process, we measured phospho-GluA2-S880 levels in cerebellar slices of wild-type and *Ptprr*^−/−^ mice. Under basal conditions, phospho-GluA2-S880 levels were comparable in cerebellar slices of wild-type and *Ptprr*^−/−^ mice (Figure 4A-B), corroborating that basal AMPAR surface levels are not altered in *Ptprr*^−/−^ cerebellar slices as demonstrated by normal properties of basal PF synaptic transmission in PCs from *Ptprr*^−/−^ mice (Figure 2C-E). As expected, chemical LTD induction resulted in an increase in GluA2-S880 phosphorylation in wild-type cerebellar slices (p < 0.05, Student’s *t*-test, n = 5; Figure 4B). Importantly, pSer880 levels remained high even after removal of the K-Glu solution for 20 min (p < 0.05, Student’s *t*-test, n = 5), indicating the expected response to LTD induction. In contrast, phospho-GluA2-S880 levels in *Ptprr*^−/−^ slices did not increase during LTD induction and washout; if anything, they slightly decreased. Compared to wild-type slices, the *Ptprr*^−/−^ slices consistently demonstrated lower phospho-GluA2-S880 ratios (F_(1,8)_ = 5.732, p < 0.04, ANOVA repeated measures), providing an explanation for the absence of LTD in PTPRR deficient PCs.

### PTPRR impacts LTD via routes other than Src-mediated GluA2 phosphorylation

Src-mediated tyrosine phosphorylation of residue 876 in GluA2 prevents subsequent S880 phosphorylation and, thereby, AMPAR endocytosis [46]. We therefore asked whether PTPRR deficiency prevents LTD by enhancing phosphorylation of the triple-tyrosine motif (3Y; positions 869, 873 and 876) in GluA2. Under basal conditions, phospho-GluA2-3Y levels in wild-type and *Ptprr*^−/−^ cerebellar slices were the same (Figure 4C-D). In wild-type slices, chemical induction of LTD decreased GluA2-3Y phosphorylation, which is in line with the enhanced GluA2-S880 phosphorylation and subsequent LTD. Interestingly, GluA2-3Y dephosphorylation in response to K-Glu treatment was much more efficient in PTPRR deficient slices (p < 0.02, Student’s *t*-test, n = 4). This argues against augmented GluA2-3Y phosphorylation as the cause for LTD impairment in PTPRR knockout slices.

**Figure 5:**
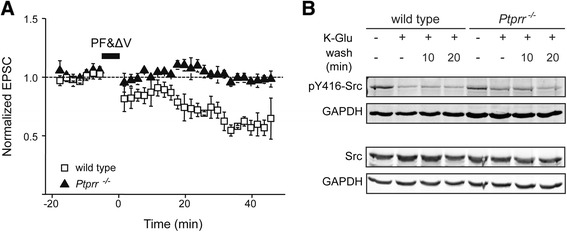
**Impairment of cerebellar LTD in** ***Ptprr*** ^**−/−**^ **Purkinje cells does not result from Src-family kinase hyperactivity. (A)** Averaged time course of PF-EPSC amplitudes triggered by PF&ΔV and normalized to their mean prestimulation level, recorded from PCs in wild-type (n = 3) or *Ptprr* ^−/−^ (n = 3) cerebellar slices while including a Src family kinase inhibitor (PP1 analog) in the patch pipet. **(B)** Wild-type and *Ptprr* ^−/−^ cerebellar slices under basal conditions,stimulated with K-Glu to induce LTD or after subsequent washes of 10 or 20 min were used for immunoblot analysis of Src kinase activity, using phospho-Y416-Src (upper panel set) and total Src (lower panel set) antibodies. GAPDH immunostaining (lower panel in each set) was used for normalization purposes. Representative images out of four independent experiments are shown.

To further substantiate this notion, we asked whether intracellular application of a specific Src family kinase inhibitor, a PP1 analog, would restore LTD in *Ptprr*^−/−^ cerebellar slices. In line with published observations [46,47], including the PP1 analog in the patch pipette solution did not affect LTD in wild-type slices (Figure 5A). The reduction of PF-EPSC was 43.6 ± 4.5%, which is not significantly different from the reduction observed in the absence of this inhibitor (Figure 2B). However, the LTD impairment observed in PTPRR-null slices was not rescued by the PP1 analog (Figure 5A, 1.4 ± 2.0%). To further exclude the contribution of aberrant Src signaling to the impairment of LTD observed in PTPRR-deficient slices, we examined phosphorylation of the autoactivating tyrosine-418 residue in Src, the mouse equivalent of chicken Src-Y416. Phospho-Y416 immunostaining declined in wild-type cerebellar slices after chemical induction of LTD (Figure 5B), in line with a previous report [48]. Basal pY416-Src levels in PTPRR deficient slices appeared mildly attenuated (Figure 5B) but this trend did not reach significance. As in wild-type samples, chemical LTD induction resulted in a decrease of pY416-Src signal, further excluding Src hyperactivity as a cause for the LTD impairment in *Ptprr*^−/−^ slices.

Collectively, our findings suggest a disturbed window of ERK1/2 signaling in *Ptprr*^−/−^ mouse brain that leads to failure of proper downstream signaling in the positive feedback loop, yielding a reduction in GluA2-S880 phosphorylation. This would be predicted to prevent AMPAR internalization from the PC surface, providing a mechanism for the impaired LTD observed in the *Ptprr*^−/−^ mouse cerebellum.

### Abnormal LTD *in vivo* in alert *Ptprr*^−/−^ mice

**Figure 6:**
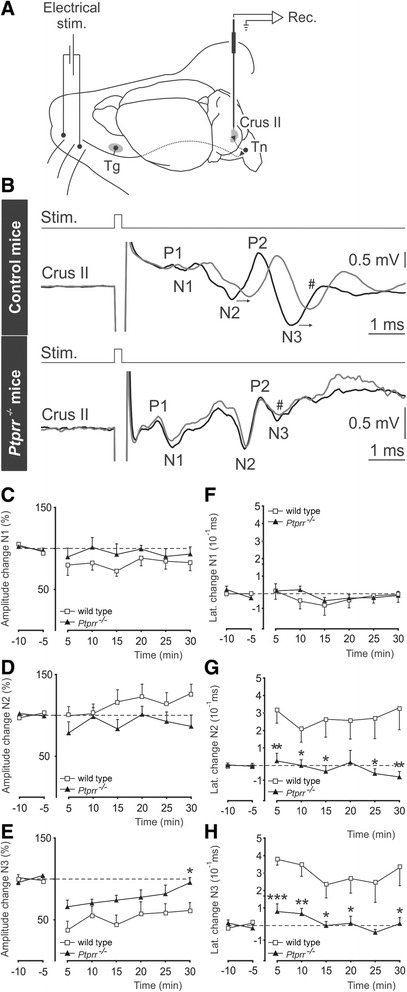
**Experimental design and LFP response to electrical stimulation of mouse whiskers and changes of cerebellar LTD in alert mutant mice. (A)** Facial dermatomes of the whisker region were electrically stimulated with a pair of needles under the skin. Sensory information comes into the Crus II area from the trigeminal nucleus (Tn) in the brainstem, which receives afferent signals from the trigeminal ganglion (Tg), **(B)** Early response associated with sensory input in the cerebellum via the trigeminal nucleus is characterized by P1-N1-N2-P2-N3 components. Averaged superimposed traces of the evoked field potential components before 8-Hz stimulation protocol, (black trace) and 30 min after 8-Hz stimulation protocol (gray trace) in control (top) and in mutant mice (bottom). Arrowheads indicate shifted latencies of postsynaptic components after 8-Hz stimulation. **(C-H)** Time course of N1, N2 and N3 amplitude **(C-E)** and latency **(F-H)** changes before (negative time periods) and after (positive time periods) the 8-Hz stimulation protocol (not shown) in 6 *Ptprr* ^*−/−*^ mice and in 6 control mice. Mean normalized data represent the peak amplitude of the different components **(C-E)** and the time difference at peak latency **(F-H)** between each 5-min interval and the mean value measured in control conditions. Significant differences between mutant and control mice are indicated with asterisks (Student’s *t*-test, *p < 0.05; **p < 0.01; ***p < 0.001).

Lastly, we wanted to assess whether the LTD abnormalities observed in cerebellar slices also occur *in vivo*. Therefore we applied to alert *Ptprr*−/− animals a recently developed LTD protocol [49,50]. We investigated local field potential (LFP) plasticity in the PC layer in response to electrical stimulation of the whisker pad before and after conditioning trains of stimulation (Figure 6A). LFP responses in alert wild-type and mutant mice demonstrated the classical P1-N1-N2-P2-N3 components, where P1-N1 reflects presynaptic input and N2 and N3 reflect the postsynaptic response at the recording site (Figure 6B, gray traces). Following 10 min of 8 Hz stimulation, the latencies of the N2 and N3 peaks increased in wild-type mice (horizontal arrows in Figure 6B). Furthermore, a marked decrease in the amplitude of the N3 component was observed (see # in Figure 6B upper trace). This effect was maximal just after conditioning (~50% amplitude decrease) and lasted for over 30 min. In contrast, no significant change in N1 or N2 amplitude was observed upon conditioning. The long-term alteration of N3 amplitude and latency thus represent read-outs of LTD in the cerebellum of alert animals [49,50].

In *Ptprr*^−/−^ mice, there were striking abnormalities in these signals. As compared to wild-type controls, the conditioning protocol had much smaller ability to attenuate the amplitude of the N3 component (see # at bottom trace in Figure 6B) and the conditioning-induced shifts in the latency of the N2 and N3 components were absent in mutant animals (Figure 6B). Mean LFP amplitude (Figure 6C-E) and latency changes (Figure 6F-H) were quantified for each of the LFP components (N1, N2 and N3) in 6 wild type and 6 *Ptprr*^*−/−*^ animals by comparing averages over each 5 min interval before and after 8 Hz stimulation. These analyses revealed reduced changes in the amplitude of the N3 component and the complete absence of N2 and N3 latency shifts. Collectively, these data implicate PTPRR as a physiological regulator of PC LTD in alert animals.

## Discussion

Cerebellar LTD requires a positive feedback loop (Figure 1) that includes MAPK as one of its key components [13]. Previous observations that *Ptprr*^−/−^ mice show increased levels of cerebellar phospho-ERK and exhibit motor coordination deficits [37] prompted us to study the role of PTPRR in cerebellar LTD. Our experiments, both in alert mice and in *ex vivo* cerebellar slices, reveal that PTPRR is required for cerebellar LTD. This requirement most likely arises from PTPRR keeping basal ERK phosphorylation levels low, thereby priming PC responsiveness to LTD-inducing stimuli.

Under basal conditions, the amount of postsynaptic AMPARs on the surface of PCs is determined by the steady-state activities of exocytotic and endocytotic transport routes [51]. Activity of PFs and CFs results in the net internalization of AMPAR from the PC surface, yielding LTD [5,6]. This effect on AMPARs is ensured by the arrangement of several key signaling molecules in a positive feedback loop, including the release of RKIP from Raf-1, resulting in the sequential activation of Raf-1, MEK, ERK1/2 and PLA2, and consequent AA production [13,16]. Elimination of an ERK1/2 negative regulator, such as PTPRR [32,37,40,41], should augment both ERK1/2 activity and AMPAR internalization. Indeed, PTPRR deficiency resulted in an increase in basal ERK1/2 activity, which would be expected to alter levels of AMPAR at the cell surface. However, our measurements revealed that basal PF-EPSC amplitudes, PPF ratios and PF-EPSC decay time constants were equal for *Ptprr*^−/−^ and wild-type PCs, indicating normal levels of synaptic AMPAR and normal synaptic transmission. Moreover, basal phosphorylation of both the endocytosis-preventing tyrosine and the endocytosis-enabling serine in the C-terminus of the GluA2 AMPAR subunit was unaffected in *Ptprr*^−/−^ cerebellar slices, indicating that AMPAR turnover occurs normally under basal conditions.

Rather than determining the basal properties of AMPARs, PTPRR appears to participate selectively in the signaling mechanism that leads to LTD induction. Despite the fact that basal ERK1/2 phosphorylation is increased in *Ptprr*^−/−^ slices, this does not lead to stronger AMPAR internalization or LTD. Rather, there is an absence of LTD in PTPRR-deficient PCs. Although a direct comparison between *in vitro* and *in vivo* LTD measurements is challenging, it is likely that we are studying the same synapses (parallel fiber to PC) in both experimental preparations. One advantage of the 8 Hz LTD-inducing protocol is that a single type of sensory input, arising from the whisker pad, produces a clear simple spike response in PCs at the latency of N3 LFP components and this is followed by a complex spike response [50]. During 8 Hz stimulation the 2 types of PC input (mossy and climbing fibers) are significantly increased [50], mimicking to some extent the *in vitro* LTD-inducing stimuli used in the present work. The close relationship between PC firing and the N3 components, the polarity inversion of this component at the PC layer level and the amplitude reduction of the N3 component by an antidromic volley applied to the parallel fiber beam strongly support the contribution of the parallel fiber-PC synapse to *in vivo* LTD [50].

Our electrophysiology data establish an absence of LTD in PTPRR deficient PCs. Concurrent with these findings, GluA2-S880 phosphorylation, which triggers AMPAR internalization, could not be induced in *Ptprr*^−/−^ cerebellar slices. Likewise, the increased basal ERK1/2 phosphorylation levels in *Ptprr*^−/−^ cerebellar slices could not be further elevated by LTD-inducing conditions: phospho-ERK1/2 levels in *Ptprr*^−/−^ and wild-type slices were identical at this point. Presumably the increased basal phospho-ERK1/2 levels reduce the range of phosphorylation that is available for maximizing ERK1/2 activity. We hypothesize that it is not the actual ERK1/2 activity levels but rather the net increase in activity levels that determines the responsiveness of PCs to LTD induction. Indeed it has been reported that enhanced ERK signaling beyond basal activity levels is required to effectively trigger LTD in cerebellar PCs [45]. Furthermore, a requirement for an increase in ERK activity - rather than the absolute level of ERK activity - to induce LTD would be consistent with the "leaky integrator" model for cerebellar LTD proposed several years ago [52]. Our current findings support and extend these notions.

In several other cell signaling studies such an ‘activity increase window’ has been proposed to explain surprising results [53,54]. For instance, neutrophils derived from rheumatoid arthritis patients show increased basal MAPK activity levels, but cytokine stimulation does not lead to a further increase in MAPK activity and this fails to elicit a physiological response [54]. The mechanism by which elevated basal MAPK activity could impair induction of effects in which they normally participate remains to be uncovered, but some understanding may come from studies in human muscle cells isolated from diabetic individuals [53]. Normally, insulin administration effectively triggers PI3K-dependent tyrosine-phosphorylation of IRS1 in muscle cells, but in cells from diabetic patients a much lower sensitivity was observed. Due to increased basal MAPK activity, a considerable portion of IRS1 was phosphorylated on its MAPK target site and this reduced its ability to be activated by PI3K [53]. Translating this to our current study, it may be that in PTPRR-deficient PCs the MAPK hyperactivity under basal conditions has directly or indirectly resulted in phosphorylation – i.e. desensitization or inactivation – of components required for AMPAR internalization. Alternatively, because PTPRR is able to dephosphorylate proteins other than MAPKs (Erkens and Hendriks, unpublished data), the absence of PTPRR activity may also impair the AMPAR regulatory machinery via increased phosphotyrosine levels of involved proteins. PTPRR has been implicated in the formation and transport of adaptin-coated vesicles in neuronal cells [55], motivating further identification of PTPRR substrates in PCs.

Could GluA2 itself be a PTPRR substrate? Efficient AMPAR endocytosis is prevented by tyrosine phosphorylation of the GluA2 subunit by Src-family tyrosine kinases [48]. An elegant study recently showed that GluD2-meditated recruitment of the protein tyrosine phosphatase PTPMEG is necessary to dephosphorylate tyrosine 876 in GluA2 and to allow subsequent serine-880 phosphorylation by activated PKC [46]. PTPMEG-null mice display impaired LTD in cerebellar PCs [56]. However, our finding of impaired LTD in *Ptprr*^−/−^ animals argues against a similar role for PTPRR. Instead, the absence of Src overactivity and GluA2-Y876 hyperphosphorylation in PTPRR-deficient PCs points to a decreased ability to serine-phosphorylate GluA2.

In addition to its role in regulating LTD induction, PTPRR may also be involved in the long-term maintenance of LTD. The observation that inhibition of MAPK activation completely blocks LTD induced by the PKC-activator TPA [13] suggests that MAPK signaling has an additional role that is independent of PKC. To maintain the late phase of LTD (>60 min after LTD-induction) the synthesis of new proteins is required [57]. It is feasible that MAPK, in addition to its central role in the positive feedback loop, also participates in signaling between active synapses and the nucleus. By entering the nucleus and phosphorylating transcription factors, such as CREB, that are required for the late phase of LTD [58], MAPK may alter the transcriptional program of the PC. By its capacity to not only dephosphorylate ERK1/2 but also to prevent its traveling to the nucleus [31,32], PTPRR thus may be able to modulate both the initial and late phases of LTD.

Despite its clear role in cerebellar LTD, it would be too simple to correlate our current findings to the disturbed motor locomotive phenotype observed in an earlier study [37]. There are indications that LTD is not essential for cerebellar learning and motor performance [59]. Moreover, backcrossing our *Ptprr*^−/−^ mice to the C57BL/6 N genetic background resulted in the loss of the overt motor defects phenotype that we previously observed in *Ptprr*^−/−^ mice on a mixed 129/C57BL/6 background (our unpublished observations), an apparent discrepancy that is more often encountered [60]. Genetic background differences also impact the behavioral phenotypes observed in these mice [37,38].

Plasticity in brain regions other than the cerebellum is also determined by processes such as long-term potentiation and LTD and these processes also rely on MAPK signaling cascades [61,62]. PTPRR is expressed in brain regions outside the cerebellum, most notably hippocampus and olfactory bulb, albeit at much lower levels [36,37,63-65]. Recently hippocampal defects were observed in *Ptprr*^−/−^ mice, including impaired novel object recognition memory [38]. In other cerebral areas, especially in the striatum, a close homolog of PTPRR, STEP, may compensate in *Ptprr*^−/−^ mice. STEP also has MAPKs as substrates [40,41,66,67] and regulates several key players involved in synaptic plasticity processes, including the AMPAR [68] within the hippocampus. The close functional similarity between PTPRR and STEP and their partly overlapping expression pattern in the brain is suggestive of partial redundancy of both PTPs in synaptic plasticity signaling processes, and warrants further investigation.

## Conclusions

We observed a significant impairment of LTD in *Ptprr*^−/−^ cerebellar PCs, both *in vitro* and *in vivo*, pointing to an essential role for the MAPK phosphatase PTPRR in neuronal responsiveness and connectivity. PTPRR activity is required for maintaining low basal ERK1/2 phosphorylation levels so that proper synaptic signals can trigger a substantial increase in MAPK activity and consequent AMPAR internalization and LTD initiation. Our findings show that PTPRR-mediated tight control of phosphorylation-mediated signaling is crucial for normal PC responsiveness. This is an example of the intricate molecular machinery that is required to fine-tune neuronal connectivity and thereby maintain proper brain function.

## Methods

### Solutions, chemicals and antibodies

The following solutions were used (chemicals were obtained from Sigma or Wako, unless otherwise specified). Standard Artificial Cerebrospinal Fluid (ACSF): 125 mM NaCl, 20 mM glucose, 2.5 mM KCl, 1.25 mM NaH_2_PO_4_, 2 mM CaCl_2_, 1.3 mM Mg_2_Cl, and 26 mM NaHCO_3_; ACSF-PTX: standard ACSF containing 0.1 mM picrotoxin (PTX); K-Glu solution: ACSF-PTX containing 50 mM K^+^ and 100 μM glutamate; pipette solution: 130 mM potassium gluconate, 2 mM NaCl, 4 mM MgCl_2_, 4 mM Na_2_-ATP, 0.4 mM Na-GTP, 20 mM HEPES (pH 7.2), and 0.25 mM EGTA; lysis buffer: 50 mM Tris–HCl (pH 7.5), 150 mM NaCl, 1% triton X-100, 100 mM NaF, 2 mM Na_3_VO_4_, 20 mM Na_4_P_2_O_7_, 1 mM PMSF and protease inhibitor cocktail (Roche Diagnostics). For some experiments, the patch pipette solution additionally contained 10 μM PP1 analog (4-amino-1-*tert*-butyl-3-(1’-naphthyl)pyrazolo[3,4-d]pyrimidine, Merck Millipore).

Primary antibodies (all from Cell Signaling Technology, unless otherwise specified) were rabbit polyclonal anti-ERK1/2 (#9102), mouse monoclonal anti-pT202/Y204-ERK1/2 (#9106), mouse monoclonal anti-MEK1/2 (#4694), rabbit monoclonal anti-pS217/S221-MEK1/2 (#9154), mouse monoclonal anti-GluA2 (MAB397, Merck Millipore), rabbit polyclonal anti-pS880-GluA2 (ab52180, Abcam), rabbit polyclonal anti-pY869/Y873/Y876-GluA2 (#3921), rabbit monoclonal anti-Src (#2109), rabbit monoclonal anti-pY416-Src (#6943), and rabbit monoclonal anti-GAPDH (#2118). For secondary antibodies, goat anti-mouse and goat anti-rabbit antibodies conjugated to IRDye680 or IRDye800 fluorescent dyes (LI-COR) were used.

### Mice

The generation of *Ptprr* knockout mice, using 129/Ola ES cells, has been described previously [37]. Founder heterozygous male mice have been backcrossed with C57BL/6 N females for 11 generations. The resulting heterozygous offspring was subsequently used for intercrossing, giving rise to *Ptprr*^*+/+*^ (referred to as wild type), *Ptprr*^*+/−*^ (heterozygous) and *Ptprr*^*−/−*^ mice. *Ptprr*^*+/+*^ mice were intercrossed to produce wild-type litters and *Ptprr*^*−/−*^ mouse crosses delivered knockout litters, which were used for the electrophysiology recordings in cerebellar slices and whole animals. The mice used for the immunoblotting experiments were established in the same fashion, but after five additional backcrosses, yielding heterozygous offspring that was intercrossed to give rise to wild-type and *Ptprr*^*−/−*^ litters. For patch clamp recordings and immunoblotting experiments, mice 14 to 21 days of age were used.

Mice were housed in a standard room with an artificial 12 h light–dark cycle (lights on at 7:00 h, lights off at 19:00 h) in Macrolon type III cages with sawdust bedding, a mouse igloo, and nest building material. Food and water were available *ad libitum* and room temperature was 21°C with controlled humidity. All procedures involving animals were approved by the Animal Care Committee of the Radboud University Medical Centre, The Netherlands, conforming to the guidelines of the Dutch Council for Animal Care and the European Communities Council Directive 2010/63/EU.

### Patch clamp recording

Whole-cell patch clamp recordings were made from PCs as described previously [13]. Sagittal slices (200 μm thickness) of cerebella from either wild-type or *Ptprr*^−/−^ mice were bathed in standard ACSF containing 0.01 mM bicuculline methochloride (Tocris). Patch pipettes (resistance 5–6 MΩ) were filled with pipette solution. Excitatory postsynaptic currents (EPSCs) were evoked in PCs (holding potential of −70 mV) by activating PFs with a glass stimulating electrode on the surface of the molecular layer (PF-EPSCs). PF-EPSCs were acquired and analyzed using LTP software (W.W. Anderson, Univ. Bristol, UK; [34]) or pClamp software (Molecular Devices). To evoke LTD by electrical stimulation (PF&ΔV), PF stimuli were paired with PC depolarization (0 mV, 200 ms) 300 times at 1 Hz. For the experiments where the PP1 analog was included in the patch pipette, LTD stimulation was applied 20–30 min after whole-cell patch clamp was made [46]. Data were accepted if the series resistance changed less than 20%, input resistance was greater than 80 MΩ, and holding current changed less than 10% during the recording. PF-EPSC decay time constant was calculated by fitting with a single exponential decay function.

### Immunoblotting

Sagittal slices (200 μm) of cerebella from wild-type or *Ptprr*^−/−^ mice were bathed at 37°C for 30 min and subsequently 1 h at RT in standard ACSF. After incubation for 20 min in ACSF-PTX, the slices were transferred to K-Glu solution for 5 min to chemically induce LTD, and if required washed with ACSF-PTX for 10 or 20 min. Slices (2 per condition) were subsequently homogenized in 100 μl lysisbuffer. Lysis was continued on ice for 30 min, followed by centrifugation for 15 min at 14.000 rpm and 4°C. Finally, the supernatant fraction was subjected to SDS-PAGE and immunoblotting according to standard procedures. Detection and quantification were performed using an Odyssey infrared imaging system (LI-COR) and accompanying software, respectively.

### Electrophysiology in alert mouse

Animals were prepared for chronic recordings of LFP [22]. Mice were anesthetized with xylidodihydrothiazin and ketamine. Animals were administered an additional dose of xylido-dihydrothiazin (3 mg/kg) and ketamine (30 mg/kg) when they demonstrated agitation or marked increases in respiration or heart rate during the procedure. In addition, local anesthesia (0.5 mL of 20 mg/mL lidocaine and adrenaline) was administered subcutaneously during the soft tissue removal. During surgery, two small bolts were cemented perpendicular to the skull to immobilize the head during the recording sessions, and a silver reference electrode was placed on the surface of the parietal cortex. To allow access to the Crus I and II areas in the cerebellum, an acrylic recording chamber was constructed around a posterior craniotomy and covered with a thin layer of bone wax.

Whisker regions were electrically stimulated with a pair of small cutaneous needles inserted under the skin (inter-electrode distance 3–4 mm; Figure 6A). Electrical stimulation consisted of a single square pulse, 0.2 ms in duration and,2 mA current intensity, delivered by an isolation unit (Isoflex, AMPI, Israel) connected to an analog pulse generator (Master 8, AMPI, Israel). The amplitude of the current was adjusted to avoid overt movements and animal discomfort. LFPs close to the PC layer and PC single-unit activity were recorded in response to single electrical stimuli delivered at semi-random intervals of 1063 s in the whisker region. An experimental session consisted of a 15-min control situation in which single electrical stimuli were given at intervals of 10 ± 3 s in the whisker region. This period was immediately followed by 10 min of 8-Hz stimulation and then by 30 min of control during which the same single stimuli were applied at the same frequency as in the control situation.

Twenty-four hours after anesthesia, alert mice were restrained for the recording session. The dura was removed over the cerebellum to expose the tissue in the recording chamber. Recordings were performed in the Crus I or II area, and the depths of the electrodes were noted. To avoid unnecessary stress for the animals and movement artifacts, recording sessions were performed in a quiet room when animals were awake and calm. LFP and unitary recordings were performed using quartz-insulated, platinum-tungsten fiber microelectrodes with outer and shaft diameters of 80 mm and 25 mm, respectively (Eckhorn system, Thomas Recordings©). LFP were filtered at 100 Hz high-pass and 10 kHz low-pass and were stored digitally on a computer after conversion with an analog–digital converter (Power 1401, CED©, Cambridge, UK). The recorded data were digitized continuously at 20 kHz. Off-line analysis and illustrations were performed with Spike2 CED software (CED©, Cambridge, UK). Electrophysiological responses to electrical stimulation in the whisker region were assessed by both the configuration of the LFP (which must show P1-N1-N2-P2-N3 components, Figure 6B) and by the identification of appropriate PC firing (modulated by spontaneous whisker movements and electrical stimulation).

### Statistical analysis

All data are presented as mean values ± SEM. The statistical significance (set at p < 0.05) of differences between groups was assessed using the independent samples Student’s *t*-test or ANOVA repeated measures. When normality could not be assumed, Mann–Whitney non parametrical test were used. Statistical procedures were performed using GraphPad Prism 4 software package or OriginPro 8.1 software.

## Abbreviations

AA: Arachidonic acid
AMPAR: α-amino-3-hydroxy-5-methyl-4-isoxazolepropionic acid receptor
CF: Climbing fiber
DUSP: Dual specificity phosphatase
EPSC: Excitatory postsynaptic current
ERK: Extracellular signal-regulated kinase
IP_3_: Inositol trisphosphate
KIM: Kinase interaction motif
LTD: Long-term depression
MAPK: Mitogen-activated protein kinase
MEK: Mitogen-activated protein kinase/extracellular signal-regulated kinase kinase
mGLuR: Metabotropic glutamate receptor
PC: Purkinje cell
PF: Parallel fiber
PKC: Protein kinase C
PLA2: Phospholipase A2
PTP: Protein tyrosine phosphatase
PTPRR: Protein tyrosine phosphatase receptor-type R
RKIP: Raf kinase inhibitory protein
